# Metabolic Syndrome in Adults Receiving Clozapine; The Need for Pharmacist Support

**DOI:** 10.3390/pharmacy11010023

**Published:** 2023-01-24

**Authors:** Kathleen Hurley, Sinead O’Brien, Ciaran Halleran, Derina Byrne, Erin Foley, Jessica Cunningham, Fionnuala Hoctor, Laura J. Sahm

**Affiliations:** 1 Pharmacy Department, Mercy University Hospital, T12 WE28 Cork, Ireland; 2 Pharmaceutical Care Research Group, School of Pharmacy, University College Cork, T12 K8AF Cork, Ireland; 3 North Lee Mental Health Services (NLMHS), T12 WE28 Cork, Ireland; 4 St Mary’s Health Campus, St Mary’s Campus, Gurranabraher, T23 TH9D Cork, Ireland

**Keywords:** metabolic syndrome, clozapine, treatment resistant schizophrenia, pharmacist

## Abstract

People who are diagnosed with treatment resistant schizophrenia (TRS) are likely to have clozapine as a therapeutic management option. There is a high prevalence of metabolic syndrome in patients receiving clozapine. To mitigate against this, monitoring of weight, waist circumference, lipid profile, glycated haemoglobin (HbA1c), fasting blood glucose (FBG) and blood pressure (BP) is recommended. The aims of this study were to examine the prevalence of metabolic syndrome and whether any variables were correlated with its development, and to highlight any opportunities for the pharmacist to offer support. This study was conducted in an urban hospital and its associated Clozapine Clinic in Cork, Ireland. A retrospective audit assessed the prevalence of metabolic syndrome using the International Diabetes Federation (IDF) criteria. Patients were eligible for inclusion if they were aged 18 years or more, registered with the Clozapine Clinic, and had the capacity to provide informed consent. All data were entered into Microsoft^®^ Excel ^®^ (Microsoft Corporation) and further statistical analysis was undertaken using R, *t*-tests, Fisher’s Exact Test and Mann–Whitney U tests as appropriate, and *p* ≤ 0.05 was considered statistically significant. Of 145 patients (32% female; mean age (SD) 45.3 (±11.7) years; 86.2% living independently/in family home), nearly two thirds (n = 86, 59.3%) were diagnosed with metabolic syndrome. The mean age of participants with metabolic syndrome was 44.4 years (SD = 10.8), similar to the 46.6 years (SD = 12.8) for those without. Variables that were identified to be statistically significantly associated with metabolic syndrome included waist circumference, weight, triglycerides, high density lipoprotein-cholesterol (HDL-C), BP, FBG and HbA1c. The high incidence of metabolic syndrome in this patient population highlights the need for continued physical health monitoring of these patients to ameliorate the risk of developing metabolic syndrome.

## 1. Introduction

Patients with schizophrenia have more than double the mortality risk compared with the general population [1], and their life expectancy is 15–25 years shorter [2]. Recent research suggests that this trend is accelerating, and a large proportion of this premature mortality is due to preventable physical health problems [3]. Antipsychotic medications, which are the cornerstone treatment for schizophrenia [4], have a large side effect profile and can induce various metabolic, cardiovascular and neuroendocrine conditions. The antipsychotic that this study focuses on is clozapine. Clozapine is a second-generation antipsychotic (SGA) that exhibits superior efficacy and effectiveness for those with treatment resistant schizophrenia (TRS).

This literature highlights that the care provided to patients who are prescribed clozapine is not seamless [5,6,7,8]. A high percentage of GPs and pharmacists are not aware that their patients are prescribed clozapine. As stated by Murphy et al., discrepancies are commonly found when outpatient psychiatric medication records are compared with primary care records, such as general practitioner (GP) prescriptions or community pharmacy medication lists [6]. In Ireland, this topic has also been reviewed by Barrett et al., and one of the main findings was that only 33.7% of community pharmacists were aware of co-prescribed clozapine [5]. A global report in 2017 by the World Health Organization (WHO) on medication safety emphasised that improving communication between primary and secondary care was vital to avoid medication-related harm [9]. Maintaining an accurate, comprehensive, and up-to-date medication list is pivotal to reducing serious medication errors [10] and allows one to check for drug-drug interactions (DDI). It is important that patients with schizophrenia have accurate medication records, as they are known to have increased vulnerability for mortality and physical co-morbidities [11]. This has a major impact on the standard of care that this patient cohort is receiving, as drug-drug interactions between clozapine and other medicines may not be highlighted, and patients presenting with physical health concerns such as constipation in the community pharmacy setting may not be considered as experiencing side effects from clozapine.

Metabolic syndrome is defined as the co-occurrence of metabolic risk factors for cardiovascular disease and type two diabetes mellitus, which include hypertension, dyslipidaemia, abdominal obesity, and hyperglycaemia [12]. There is a high prevalence of metabolic syndrome in patients receiving clozapine [13]. Research recommends more robust monitoring of weight, waist circumference, lipid profile, HbA1c, fasting blood glucose and blood pressure at clozapine clinics to prevent consequent health complications [14]. The aim of this study was to examine the prevalence of metabolic syndrome in these patients using the International Diabetes Federation (IDF) [15] criteria, as well as to examine whether any variables were correlated with the development of metabolic syndrome and to highlight any opportunities for the pharmacist to offer support. 

Objectives:

To examine the demographics of patients prescribed clozapine, and their reported co-morbidities, physical health characteristics, co-medications, drug-drug interactions (DDI) and side effects.

To assess the prevalence of metabolic syndrome in this cohort and whether any variables were correlated with the development of metabolic syndrome.

## 2. Methods

Permission for conducting this research study was granted by the Clinical Research and Ethics Committee (CREC) of the Cork Teaching Hospitals. This study was conducted at an urban hospital and its associated Clozapine Clinic in Cork, Ireland. Patients who are prescribed clozapine have routine blood taken by Clozapine Clinical Nurse Specialists (CCNS). Patients receiving clozapine were invited to participate in this study by the Research Pharmacist K Hurley (KH). This was done in the Clozapine Clinic when patients came to collect their clozapine. Patients were then assessed for eligibility; inclusion criteria included all patients being aged 18 years or over and registered with the Denzapine^®^ Patient Monitoring Service (DPMS) [16]. Exclusion criteria included those who did not attend the clinic during the study period (24 May 2021 to 21 June 2021), or those unable to provide informed consent. If they agreed to take part in this study, the informed consent process was conducted prior to any data collection.

The data contained within the medical notes, and/or obtained by contacting the patient’s community pharmacist, regarding the following variables were manually extracted: age, gender, weight, waist circumference, blood pressure, total daily dose of clozapine, smoking status (ever/never), living arrangements (independent/family home or other (nursing home/hostel)), number of years taking clozapine as recorded on the DPMS^®^ website, co-morbidities, and concomitant medications. In addition, the following blood results were obtained from iLab APEX^®^Laboratory Information Management System (v.5.8) supplied by DXC Technology PLC (the hospital laboratory software system): triglycerides, HDL, LDL, fasting plasma glucose, haemoglobin A1c (HbA1c), prolactin, serum clozapine and norclozapine levels. BMI, weight, waist circumference, BP and smoking status results were within 6 months, and glucose regulation, blood lipids and prolactin results were within 12 months as per the Mental Health Commission guidelines [17].

KH undertook a DDI check between clozapine and all medicines, using Lexicomp [18] and Stockley’s Interaction Checker (SIC) [19]. In community pharmacies in Ireland, pharmacists would routinely use SIC [19]. However, in the hospital setting, the pharmacists would have access to both SIC and Lexicomp [18]. A previous study based in Cork University Hospital used both interaction checkers and found some discrepancies between recommendations; therefore, we thought it prudent to check both [5].

DDIs were classified under three categories:

1. Avoid Combination: Contra-Indicated (CI)/Life-Threatening
2. Consider Therapy Modification/Dose Adjustment
3. Monitor Therapy

KH calculated the presence or absence of metabolic syndrome manually, using the IDF criteria.

To ensure confidentiality, the data generated during the study were coded. Data that directly identified the participant (uncoded data) was stored in the medical files at the clinical site in which the data were obtained. A ‘key’ linking the participants study number to their name was stored securely, within a locked cabinet in a room with restricted access, by KH. All data were saved on a password-encrypted laptop. The following table illustrates the IDF criteria for metabolic syndrome diagnosis (Table 1).

Data Analysis

All data obtained from the audit were entered into Microsoft Excel (IBM Corporation) for descriptive statistics. Further statistical analysis was undertaken using R, T-tests, Fisher’s Exact Test and Mann–Whitney U tests as appropriate, and a *p* ≤ 0.05 was considered statistically significant.

## 3. Results

Of a total of 154 patients registered for clozapine therapy in this study period, one patient died, two patients were deemed too unwell to be asked to participate and three patients discontinued clozapine therapy. Of the remaining 148 patients, three patients did not agree to participate, leaving 145 patients who provided written informed consent.

The demographics of the participants are shown in Table 2. The clozapine indication for all but two patients was TRS, and one patient was prescribed clozapine for psychotic disorder in Parkinson’s Disease.

Metabolic Syndrome

The prevalence of metabolic syndrome was 59.3% (n = 86) using the IDF [20] criteria. The mean age of participants with metabolic syndrome was 44.4 years (SD = 10.8), similar to 46.6 years (SD = 12.8) for those without metabolic syndrome.

Waist Circumference

Of 145 patients, 115 had central obesity corresponding to 41 females (WC ≥ 80 cm) and 74 males (WC ≥ 94 cm).

Type 2 Diabetes Mellitus

The prevalence of Type 2 diabetes mellitus (T2DM), based on documentation in the medical notes or associated drug treatment, was 11.7% (n = 17). The scientific laboratory marker used by the clozapine clinic for T2DM is HbA1c_;_ see Table 3 for the breakdown.

There were three patients where a level > 48 mmol/mol was measured but neither diagnosis nor treatment was in place.

In the case of those where T2DM was documented in the medical notes and/or patients were prescribed anti-hyperglycemic agents (n = 17), eight patients were well controlled with HbA1c < 48 mmol/mol, five had elevated levels between 48–53 mmol/mol, and four had levels > 53 mmol/mol.

Hypertension

The thresholds for the groups were >140/90 mmHg and 130/80–140/90 mmHg and the prevalence of hypertension, based on documentation in the medical notes or associated drug treatment, was 9.7% (n = 14). Seven patients had blood pressure readings > 140/90 mmHg. One of these seven was prescribed anti-hypertensive medication. Thirty-three patients (22.8%) had blood pressure readings > 130/80 mmHg and of these, six patients were prescribed anti-hypertensives.

Hypercholesterolemia/Dyslipidaemia

A total of 46 patients (31.7%) had a documented diagnosis of dyslipidaemia and/or were prescribed lipid-lowering agents. There is no natural cutoff between normal and abnormal cholesterol levels. For adults, a total cholesterol level of less than 5 mmol/L is desirable, but lower values have shown to be more cardioprotective [22]. Over 50% (79/145) of patients had a total cholesterol of over 5 mmol/L, but of these only 29.1% (23/79) were receiving medication to manage hypercholesterolemia (Table 4).

The total cholesterol level is only a guide to the risk of atherosclerosis. Low-density lipoprotein cholesterol (LDL-C) levels are the primary therapeutic target, and a summary of results can be seen in Table 5. LDL-C levels of <3.0 mmol/L are desirable, but lower values (<2.0 /L) are more cardioprotective, and recommended, depending on the cardiovascular risk factors associated [22] (Table 5). Of those, thirty-four (73.9%) patients were well controlled with LDL-C < 2.6 mmol/L, while 26.1% (n = 12) had documented LDL > 3.0 mmol/L. Less than half (46.9%) of 68 patients had an LDL-C > 3.0 mmol/L, of whom seven were receiving medication.

The frequency and type of co-morbidities of participants is shown in Table 6.

Drug-Drug Interactions (DDI)

In addition to clozapine, patients (n = 116) were prescribed a total of 615 medicines. The mean number of co-prescribed medicines (not including clozapine) per patient was 4.2 (SD ± 3.6) and ranged from zero to 13.

Medicines for each patient were checked for drug-drug interactions (DDI) with clozapine using two separate reference sources: Lexicomp^®^ and Stockley’s Interaction Checker (SIC). Using Lexicomp^®^, it was found that 58.3% (359/615) of medicines had documented interactions with clozapine; 54.3% (334/615) were found using SIC. For those patients taking co-prescribed medicines (n = 116), Lexicomp^®^ recorded 3.1 DDIs per patient and SIC recorded 2.9 DDIs per patient.

***Avoid Combination: Contra-Indicated (CI)/ Life-Threatening DDIs***

Using Lexicomp^®^, the identified prescribed drugs contra-indicated with clozapine were amisulpride and inhaled anti-muscarinic agents, and domperidone was identified using SIC (Table 7).

Table 8 lists the frequency and type of side effects experienced by patients prescribed clozapine. Less than a third (29.7%, n = 43) reported no side effects. Over half (59.3%, n = 86) of patients reported the presence of hyper-salivation, while 27.6% (n = 40) suffered from sedation or drowsiness.

Those variables that were identified to be statistically significantly associated with metabolic syndrome after univariate analyses included waist circumference, serum clozapine, serum norclozapine, current weight, triglycerides, HDL-C, diastolic and systolic blood pressure, fasting plasma blood glucose and HbA1c. Table 9 contains the results of the univariate analysis.

## 4. Discussion

Over half of this patient population (n = 86, 59.3%) were diagnosed with metabolic syndrome, twice the prevalence of the general Irish population [23]. More recent Irish data from the Irish Longitudinal Study on Ageing (TILDA) have demonstrated that two in five of the 1.2 million population aged 50 years or more meet either the ATPIII or IDF criteria [24]. Increasing age, male sex, lower educational attainment, and lower physical activity were all associated with an increased likelihood of metabolic syndrome. This correlates with the literature, in which the percentage of participants with metabolic syndrome ranged from 46% to 61.6% [13,25,26,27,28,29]. Several variables emerged as statistically significant (*p* < 0.05) with the presence of metabolic syndrome, including increased waist circumference, weight, triglycerides, blood pressure, fasting plasma glucose, HbA1c, serum clozapine and norclozapine levels and decreased HDL-C. The high prevalence of metabolic syndrome in this patient population highlights the long-term health risks associated with clozapine treatment for this cohort. It has been shown that patients with metabolic syndrome have a two- to threefold increase in cardiovascular disease mortality [30]. Therefore, these data emphasize the opportunity that pharmacists, in the community or hospital, may offer for increased screening for metabolic syndrome in patients prescribed clozapine. The screening process should ideally include fasting blood glucose and lipids, HbA1c, blood pressure, body weight and waist circumference.

In this study, more than two-thirds of the participants were male, in line with the literature where schizophrenia is generally reported to be higher in males than in females [31]. This is also broadly in line with the gender distribution in other studies [5,6]. The average age of participants was 45 years, which was similar to the two Irish studies by Barrett et al. and Ahmed et al. [5,13]. Participants were taking a mean dose of 315 (±126) mg of clozapine per day; this is slightly lower than other Irish studies. Barrett et al. quoted a mean dose of clozapine of 350 (±136) mg [5], while the study conducted in the West of Ireland stated a mean clozapine dose of 414.7 (±151.1) mg for patients diagnosed with metabolic syndrome and 377.5 mg (±175.2) mg without metabolic syndrome [13]. However, compared to international data, the mean dose of clozapine was lower in other countries. In a study conducted in China, it was found that 193.8 (±95.4) mg [32] was used, and a study in Italy found an average of 250.46 (±113.22) mg [25]. This may be an indicator that clozapine treatment is used at an earlier stage of therapy in these countries, due perhaps to differing clinical guidlines. In this present study, more than half the participants had been taking clozapine for ten years or more. This is comparable to other studies; Barrett et al. states that almost half (49.1%) of participants were prescribed clozapine for ten years or more [5]. The majority of patients were residing in the community, which is similar to other studies [5,26,33]. Most participants were non-smokers (60%), in line with two studies [5,33] but in contrast with a Chinese study by Zhang et al. and an Irish paper by Ahmed et al., in which over 60% of participants were smokers [13,32].

Increased waist circumference and weight was a common risk factor for metabolic syndrome that resonated throughout the literature [13,26,27,28,29,32,34], and this study echoed this finding. Interestingly, one study demonstrated that self-reported moderate physical activity protected patients against metabolic syndrome [33]. It would be interesting to do further study on patients’ BMI and/or weight. Ahmed et al. examined weight prior to initiation of clozapine treatment, and current weight. This study concluded that those with metabolic syndrome gained a significantly larger amount of weight since commencing clozapine treatment than those without metabolic syndrome [13].

Poorly controlled T2DM, increased fasting plasma glucose and elevated HbA1c were reviewed in four studies [13,25,32,33], with authors agreeing that these factors statistically increased the risk of metabolic syndrome. Our study reiterates this finding, as higher fasting plasma glucose levels and HbA1c were significantly associated with the presence of metabolic syndrome. More than one in ten patients in this study had a diagnosis of T2DM; whilst this is lower than other studies, which reported almost double this [5,35], it would seem that some patients in our study with high HbA1c may not have had a formal diagnosis. HbA1c was measured at >53 mmol/mol in over four of those with documented T2DM. This indicates poorly controlled T2DM, and perhaps poor medication adherence, adding to the risk. Three patients who were not formally diagnosed with T2DM had HbA1c readings greater than 53 mmol/mol. Therefore, in this patient cohort, there is the possibility that if additional screening were offered, more patients would be diagnosed with T2DM. There is an opportunity to educate patients on nutrition, exercise, lifestyle and medication adherence. Pharmacists have the knowledge to provide individual advice, optimise and support adherence, and advise on appropriate blood glucose monitoring, while being aware of the side effect profile of clozapine. In one feasibility study, the community pharmacist successfully delivered screening of diabetes and cardiovascular disease (CVD) risk [36]. However, to the best of our knowledge, pathways for the integration of a pharmacist-delivered screening service within the Health Service Executive (HSE) in Ireland are not in place, and would require further research and exploration.

The lipid profiles of patients who were diagnosed with metabolic syndrome were examined, and findings highlighted that increased triglycerides and lower HDL-C were both significantly associated with the presence of metabolic syndrome. Similarly, the literature identified that metabolic syndrome was associated with a higher prevalence of hypertriglyceridemia and lower HDL-C [25,26,27,28,32]. More than half the patients in this study had a total cholesterol of over 5 mmol/L, in line with Barrett et al. [5]. In our study, more than two in five patients had an LDL-C > 3.0 mmol/L; however, only 10% were prescribed medication for this indication. This highlights the need for regular monitoring of this variable, and increased medication interventions to reverse this cardiac risk factor. Community pharmacists are recognised as an accessible healthcare professional in the Irish healthcare system, and in healthcare systems worldwide [37,38]. In 2016, a survey conducted by the Pharmaceutical Society of Ireland (PSI), the Irish pharmacy regulator, found that a third of a million people visited a community pharmacy in Ireland weekly, and that almost three in five of those aged 16 years and older visited their community pharmacy more than once a month [39]. On the other hand, a survey conducted by Healthy Ireland in 2019 found there was an average of 4.5 GP visits per person per annum in those aged 15 years and older [40]. Additionally, the IPSOS Veracity poll 2022 placed community pharmacists as the second highest most trusted profession, with 93% of respondents stating that they trust pharmacists to tell the truth [41]. Community pharmacists are thus well-placed to recognise and interact with patients who are receiving clozapine therapy. Therefore, we postulate that a pharmacist would be an ideal healthcare professional to coordinate and advise on medication to control cholesterol, and monitor blood results, while being aware of the side effect profile of clozapine and the associated risks.

The prevalence of hypertension was estimated at just below 10%, which compares well to a UK study conducted in 2020 [14]. Higher levels of diastolic and systolic blood pressure were both significantly associated with the presence of metabolic syndrome. From the literature, Bai et al. demonstrated that an increased BMI after initiation of clozapine treatment was a significant factor for elevated blood pressure [34], while Brunero et al. stated that the emerging predictive model of metabolic syndrome retained increased blood pressure as a factor also [28]. Many community pharmacists are trained in monitoring blood pressure and are the medication experts for prescribing anti-hypertensive medications.

Clozapine and norclozapine levels in patients with metabolic syndrome were significantly higher compared with those without metabolic syndrome. This correlation has been reviewed in the literature and the results are debated, as plasma concentration, rather than clozapine daily dose, seems to have an impact on metabolic changes [42]. One study confirms a high prevalence of metabolic side effects with clozapine and suggested higher clozapine level and certain pharmacogenetic markers as important predictors of metabolic syndrome [43].

According to the product licence, no clozapine–drug combination is absolutely contraindicated [30], but some medications may pose less risk when used concurrently with clozapine. We found that for patients (n = 116) co-prescribed other medicines, DDIs occurred in 72.4% (n = 84) of patients. Our figure is slightly lower compared with the literature. Barrett et al. demonstrated DDIs in 96.9% (n = 94) of patients co-prescribed medications, while Leung et al. reported 90.4% (n = 94) [5,44]. The reason for this is not understood. However, the participants that were included in the Leung et al. study were all hospitalised; this would indicate that they were more unwell and prescribed more medication, and were more at risk of a DDI. There were, on average, 3.1 DDIs per patient using Lexicomp^®^ and 2.9 using SIC, and this was in line with Barrett et al. figure of 2.9 DDIs per patient using Lexicomp^®^ and 2.5 using SIC. Using Lexicomp^®^ (Lexicomp Online. Waltham, MA: UpToDate, Inc.) 58.3% (359/615) of medicines had documented interactions with clozapine, compared with 54.3% (334/615) using SIC, which is comparable to Barret et al., who found that 53.5% of patients were exposed to a major or moderate interaction as per Lexicomp^®^ and 58% exposure as per SIC.

The medications listed as contraindicated or life-threatening using Lexicomp^®^ were amisulpride and inhaled anti-muscarinic agents. Eighteen patients were prescribed amisulpride; Lexicomp^®^ did not recommend this due to the risk of neuroleptic malignant syndrome. Augmenting clozapine with amisulpride is already a strategy commonly used by clinicians in practice, without robust evidence based on the risks and benefits [45]. The 2014 NICE (National Institute for Health and Care Excellence) guideline on the prevention and management of psychosis and schizophrenia in adults endorses that a second antipsychotic should be prescribed to augment treatment with clozapine for people whose illness has not responded sufficiently to clozapine on its own [46]. However, it does not name a specific drug to use; rather, it recommends that healthcare professionals should choose one that does not worsen the side effects associated with clozapine. One case study demonstrates that the interaction between these two medications may be sequence dependent. In this scenario, clozapine was commenced after the amisulpride was initiated. Within days, the patient presented with severe akathisia, restlessness, hypertonia along with sialorrhea, and tachycardia [47]. Withdrawal of clozapine did not remit akathisia and agitation completely; this led to a hypothesis that it may not be clozapine per se, but its interaction with amisulpride, that is instrumental in the generation of the akathisia symptoms. This study queried whether the sequence of introduction of medications may have some role in this augmentation strategy, as clearly it is safer to augment a partial responder with an optimal dose of clozapine with amisulpride than the reverse, because there are more chances of producing side effects in the latter schedule [47]. This demonstrates the need for more research and guidelines on this topic.

On reviewing the notes, less than a third of participants stated that they experienced no side effects associated with clozapine. Nearly 60% (n = 86) of patients suffered from hypersalivation. The product licence for Denzapine^®^ states that this side effect is very common, i.e., ≥ 1/10 [30]. The literature states that anywhere from 30–90% of patients report hypersalivation [48]. Ten patients (6.8%) were prescribed hyoscine hydrobromide to counteract this side effect; however, per their medical notes, hypersalivation was still present. Worryingly, out of the ten patients who were prescribed hyoscine hydrobromide, only one patient was prescribed a laxative to counteract the anticholinergic effects of this agent. This is an example of how a clinical pharmacist may have an impact on the quality of life for patients who are prescribed clozapine. Patients may not be prescribed the optimal dose or medication, and behaviour modifications could be adapted, such as chewing gum to promote swallowing.

Sedation or drowsiness occurred in over a quarter of patients (n = 40). This is in line with the product licence for Denzapine^®^, which states that this side effect is very common, i.e., ≥1/10 [30]. Suggestions to counteract this side effect may include dose reduction, administration of most or all of the clozapine dose at night and avoiding other sedating medications. Constipation, which is a common side effect of clozapine, can have serious consequences [49]. The incidence for gastrointestinal hypomotility has been reported to be 4 to 8 per thousand, and, according to the literature, has a case fatality of 15% to 27.5% [11]. However, in 2016, a systematic review and meta-analysis was carried out on the prevalence and predictors of clozapine-associated constipation [50]. Shirazi et al. established a pooled prevalence of clozapine-associated constipation of 31.2% [50]. In our study, 12.4% (n = 18) of the participants reported constipation, and only 3 of these 18 patients had laxatives prescribed by their GP. This may be underreported, as this condition is self-managed in many cases. Awareness of this important side effect needs to increase, because early detection and management is vital to minimizing risk. In addition, two patients in this study were prescribed a codeine containing combination product and neither patient was prescribed a laxative. Community pharmacists play a key role in the pharmaceutical management of bowel problems, recommending over-the counter (OTC) and prescription medicines to patients. Therefore, they would be an ideal HCP to lead a team approach to enhance patient monitoring, treatment, and improved guidelines to prevent clozapine induce hypomotility.

Pharmacogenomic testing for specific genes associated with the pharmacodynamic action, metabolism or clearance of antipsychotics may offer an opportunity to personalize the therapeutic regimen of those with these mental health conditions. Pharmacogenomic testing has previously been shown to reduce the incidence of adverse drug reactions (ADR) and improve clinical outcomes in patients with TRD [48].

Pharmacogenetic testing in those with psychosis may allow determination of patients who are at higher risk of weight gain or those who are more likely to benefit from treatment with antipsychotics [49,50]. There are also some specific circumstances in which it is advised that the dosing of antipsychotics, such as aripiprazole, needs to be revised based on pharmacogenetic variants. This [50] is an area for future research. Further epidemiological studies are required to investigate the long-term impact of clozapine on mortality and morbidity in schizophrenia. This study highlights the possibility for pharmacists to support patients by providing additional opportunities for physically monitoring this patient population. Such initiatives could include improvement in lifestyle-related behaviours (exercise, smoking habits, diet) and adhering to medication management of lipid and glucose dysregulation and elevated blood pressure where indicated. Approaches using interprofessional models of care, which include pharmacists specialising in psychiatric care, could help meet the needs of patients receiving clozapine.

### Limitations

This is a single-site study, and clozapine services differ from country to country, which limits the generalisability of the findings. The sample size may have been small, and when studying the prevalence of metabolic syndrome, it was not possible to have a control group. The study did not directly assess the extent of symptom severity among patients, or indeed whether patients had been diagnosed with metabolic syndrome prior to initiation of clozapine therapy. Other parameters that may have played a role were not included, such as physical activity level, dietary habits, and psychotic and affective symptoms. Further research should address these issues in more detail and in larger cohorts.

## 5. Conclusions

This study confirms the high prevalence of metabolic syndrome for patients receiving clozapine, and identified increased waist circumference, weight, serum clozapine and norclozapine, triglycerides, blood pressure, fasting plasma glucose, and HbA1c and decreased HDL-C as variables associated with the syndrome. This study highlights the opportunity for pharmacists to provide additional physical health checks and monitoring the side effect burden for patients who are prescribed clozapine. A pharmacist in the community or as part of a multi-disciplinary mental health team could facilitate interventions to offset the increased risk of developing metabolic syndrome in patients who are prescribed clozapine, thus further optimising their care.

## Figures and Tables

**Table 1:** IDF criteria for diagnosis of metabolic syndrome.

Central Obesity: BMI > 30 kg/m^2^ or WC ≥ 94 cm (Male) or ≥ 80 cm (Female)	
Insulin resistance ^a^	Raised fasting glucose (≥5.6 mmol/L)
Blood Pressure	SBP ≥ 130 mmHg or DBP ≥ 85 mmHg, or treatment
Triglycerides	≥1.7 mmol/L or treatment ^b^
High Density Lipoprotein	≤1.03 mmol/L (male) or ≤1.29 mmol/L (female)

Notes: IDF—International Diabetes Foundation; WC—waist circumference; BMI—body mass index; SBP—systolic blood pressure; DBP—diastolic blood pressure. ^a^ Diagnosis of diabetes, treatment of diabetes or HbA1c ≥ 39 mmol/mol (5.7%) used as surrogate for raised fasting glucose; ^b^ Fibrates or Nicotinic acid.

**Table 2:** Study participant demographics.

Study Participant Demographics	Variable	N	%
Gender	Male	98	68
	Female	47	32
Age (Years)	Mean (SD)	45.3 (±11.7)	-
	Median	43.8	-
Daily dose of clozapine (mg)	Mean (SD)	315 (±126)	-
	Median	300	-
Duration Prescribed clozapine (years)	<0.5	0	0
	≥0.5–1	7	4.8
	>1–5	28	19.3
	>5–10	30	20.7
	>10	80	55.2
Clozapine Indication	Treatment resistant schizophrenia	143	98.6
	Psychotic disorder in Parkinson’s disease	1	0.7
	Not specified	1	0.7
Current Smoking Status	Smoker	56	38.6
	Non-Smoker	89	61.4
Living Arrangements	Independent/ Family Home	125	86.2
	Other (Nursing Home/ Hostel)	20	13.8

SD = standard deviation; - = not applicable.

**Table 3:** HbA1c values for the study participants.

HbA1c (mmol/mol)	n = 145	%	No. of Patients Receiving Antidiabetic Medication to Manage T2DM
<42	116	80.00	4
43–47	17	11.72	4
48–53 *	5	3.45	5
>53 *	7	4.83	4

* Levels > 48 mmol/mol confirm T2DM [21].

**Table 4:** Total Cholesterol (TC) Levels (mmol/L) of participants.

TC (mmol/L)	N = 145	%	No. of Patients Receiving Medication to Manage Hypercholesterolaemia
**<5**	66	45.52	23
**5–6**	51	35.17	17
**>6**	28	19.31	6

**Table 5:** Low-density lipoprotein cholesterol (LDL-C) levels (mmol/L) of participants.

LDL-C (mmol/L)	n = 145	%	No. of Patients Receiving Medication to Manage Hypercholesterolaemia
<2.6	62	42.76	34
2.6 - < 3.0	15	10.34	5
3.0 - < 4.9	65	44.83	7
≥ 4.9	3	2.07	0

**Table 6:** Frequency and type of co-morbidities of participants (n = 145).

Co-Morbidities of Participants	n *	%
Hypercholesterolemia/ Dyslipidaemia	46	31.7
Type 2 Diabetes Mellitus	17	11.7
Hypothyroidism	17	11.7
Hypertension	14	9.7
Gastrointestinal (Constipation) (GORD ^#^/dyspepsia) (Both)	40 7 25 8	27.6 4.8 17.2 5.5
Respiratory (Asthma & COPD ^~^)	17	11.7
Cardiovascular Disease	11	7.6
Tachycardia	17	11.7

* Patients may have multiple co-morbidities; ^#^ = GORD gastro oesophageal reflux disease; ^~^ COPD = chronic obstructive pulmonary disease.

**Table 7:** Co-prescribed contra-indicated (CI)/ life-threatening medicines with clozapine.

Contra-Indicated (CI)/ Life-Threatening DDIs		
Medicine	**Lexicomp^®^ (n = 30)**	**SIC (n = 2)**
Amisulpride	19	-
Domperidone	-	2
Inhaled Anti-Muscarinic Agent	11	-

Side-effect profile.

**Table 8:** Frequency and type of side effects of participants (n = 145).

Side Effect	n = *	%
No side effects	43	29.7
Sedation/Drowsiness	40	27.6
Hyper-salivation	86	59.3
Dizziness	16	11.0
Constipation	18	12.4
Nausea/Vomiting	5	3.4
Perspiration	9	6.2
Dry mouth	16	11.0
Urinary problems	13	9.0
Tremor	11	7.6
Visual disturbance	6	4.1

* Patients may have multiple side effects.

**Table 9:** Univariate results for clinical variables in subjects with (n = 86) and without (n = 59) metabolic syndrome using the IDF criteria for metabolic syndrome.

Variables	With Metabolic Syndrome (n = 86), n (%)	Without Metabolic Syndrome (n = 59), n (%)	*Fisher Test*	*p*-Value (*p* < 0.05 Considered Statistically Significant)
Gender Male Female	57 (58.2%) 29 (61.7%)	41 (41.8%) 18 (38.3%)	0.864	0.7211
Current Smoker	33 (38.4%)	23 (40.0%)	0.975	1
Taking a concomitant antipsychotic drug	41 (47.7%)	26 (44.1%)	1.155	0.7356
Taking other medications	69 (80.2%)	47 (79.7%)	1.036	1
Waist Circumference ≥ 94 cm men & ≥ 80 cm women	78 (90.1%)	29 (49.2%)	9.241	**1.153 × 10^7^**
Variables	**Mean (SD)**	**Mean (SD)**	***t*-test**	***p*-value**
Age	44.4 (10.8)	46.6 (12.8)	1.074	0.2853
Duration of clozapine treatment (years)	9.3 (5.1)	9.5 (5.0)	0.221	0.8251
Clozapine dose (mg/day)	305.4 (118.0)	328.1 (137.5)	1.034	0.3034
Serum Clozapine (mg/L) (0.35–0.60 mg/L)	0.44 (0.18)	0.34 (0.15)	−3.545	**5.34 × 10^4^**
Serum Norclozapine (mg/L)	0.24 (0.11)	0.20 (0.08)	−2.813	**5.6 × 10^3^**
Current weight (kg)	100.7 (19.5)	83.2 (17.8)	−5.579	**1.32 × 10^7^**
LDL-C (mmol/L)	2.8 (1.0)	3.1 (1.0)	1.581	0.1163
Blood pressure, systolic (mmHg)	128.2 (13.7)	117.7 (11.9)	−4.908	**2.61 × 10^6^**
Blood pressure, diastolic (mmHg)	87.4 (7.9)	81.8 (8.1)	−4.102	**7.41 × 10^5^**
Variables	**Mean (SD)**	**Mean (SD)**	**Mann-Whitney U**	***p*-value**
Triglycerides (mmol/L) (0.3–1.7 mmol/L)	2.4 (1.1)	1.3 (0.7)	827.5	**6.04 × 10^12^**
HDL-C (mmol/L)	1.2 (0.3)	1.5 (0.4)	3805.0	**3.36 × 10^7^**
Fasting plasma glucose (mmol/L) (3.9–5.6 mmol/L)	6.8 (2.4)	5.3 (0.5)	1160.0	**2.91 × 10^8^**
HbA1c (mmol/mol) (20–42 mmol/mol)	42 (11.1)	36.8 (4.5)	1692.5	**6.59 × 10^4^**
Prolactin (mIU/L) Female 102–496 mIU/L Male 86–324 mIU/L	362.5 (384.5)	392.5 (570.5)	2440.5	0.6992

Statistically significant results are highlighted in **bold.**

## Data Availability

Not available.
